# Early recognition of eosinophilic granulomatosis with polyangiitis in outpatient settings: musculoskeletal and gastrointestinal clues beyond respiratory disease

**DOI:** 10.3389/fimmu.2026.1964154

**Published:** 2026-10-05

**Authors:** Ernesto Aitella, Massimo De Martinis, Lia Ginaldi

**Affiliations:** 1 Department of Life, Health and Environmental Sciences, University of L’Aquila, L’Aquila, Italy; 2 Allergy and Clinical Immunology Unit, Center for the Diagnosis and Treatment of Osteoporosis, Teramo, Italy; 3 Long-Term Care Unit, “Maria SS. dello Splendore” Hospital, Giulianova, Italy; 4 UniCamillus-Saint Camillus International University of Health Sciences, Rome, Italy

**Keywords:** arthralgia, biologic therapy, early manifestations, eosinophilia, eosinophilic gastrointestinal disorders, eosinophilic granulomatosis with polyangiitis, myositis, outpatient care

## Abstract

Eosinophilic granulomatosis with polyangiitis (EGPA) is a multisystem immune-mediated disease characterized by adult-onset asthma, eosinophilic inflammation, and necrotizing vasculitis of small-to-medium-sized vessels. Diagnosis is frequently prompted by clinically overt neurological, cardiac, renal, cutaneous, or gastrointestinal involvement. However, potentially informative manifestations may already be recognizable earlier, particularly when asthma, chronic rhinosinusitis with nasal polyps, and blood eosinophilia coexist with otherwise nonspecific extrapulmonary findings. Most cohorts describe manifestations at diagnosis or during established disease and provide limited information on their early temporal appearance across different specialties. This gap is particularly relevant to arthralgia and myalgia, which are common but poorly characterized, and to eosinophilic gastrointestinal manifestations that may initially be classified as organ-restricted eosinophilic gastrointestinal disorders. Biopsy-proven myositis is rare but has occasionally been reported at presentation or before clinically recognizable neuropathy. This review examines early and potentially recognizable manifestations of EGPA across outpatient settings, with particular attention to musculoskeletal symptoms, objective muscle involvement, eosinophilic gastrointestinal disease, and the therapeutic implications of distinguishing organ-restricted eosinophilic disease from systemic EGPA.

## 1 Introduction

Eosinophilic granulomatosis with polyangiitis (EGPA), formerly termed Churg–Strauss syndrome (CSS), is a rare systemic disease characterized by eosinophil-rich inflammation, necrotizing vasculitis predominantly involving small-to-medium-sized vessels, and a close association with asthma and chronic upper-airway disease (1). EGPA is classified among the anti-neutrophil cytoplasmic antibody (ANCA)-associated vasculitides, although ANCA are detectable in only a subset of patients and are predominantly directed against myeloperoxidase (MPO). MPO-ANCA-positive disease is more frequently associated with vasculitic manifestations, whereas ANCA-negative disease more often shows eosinophilic tissue-infiltrative features; however, these patterns substantially overlap and should not be regarded as mutually exclusive categories (2, 3). Type 2 immune responses, particularly interleukin-5 (IL-5)-driven eosinophil expansion and tissue recruitment, contribute to the eosinophilic component of EGPA, whereas MPO-ANCA can promote neutrophil activation and endothelial injury (4). Eosinophilic inflammation and vasculitis may therefore coexist within the same organ and patient.

The natural history of EGPA has traditionally been described as comprising a prodromal allergic/respiratory phase, an eosinophilic phase characterized by peripheral and tissue eosinophilia, and a vasculitic phase with systemic organ involvement (5). This background remains clinically useful, although the phases frequently overlap, may occur in a non-linear sequence, and are not invariably distinguishable in individual patients. Clinically informative manifestations may therefore become apparent before classical vasculitic features are recognized.

EGPA is often diagnosed after highly suggestive findings such as mononeuritis multiplex, palpable purpura, cardiomyopathy, glomerulonephritis, or severe gastrointestinal involvement appear. Nevertheless, potentially informative findings may have been present earlier. Patients may be followed separately for severe asthma, recurrent nasal polyposis, eosinophilia, abdominal symptoms, arthralgia, myalgia, skin abnormalities, or sensory complaints. Most reviews describe the overall EGPA phenotype, whereas fewer studies reconstruct the early clinically recognizable course, including when and how different manifestations are encountered across outpatient specialties (6).

This review therefore focuses on manifestations that may increase suspicion of EGPA before the disease becomes diagnostically obvious. Particular attention is given to musculoskeletal symptoms and rare myositis, eosinophilic gastrointestinal disease, and therapeutic choices that may differ substantially when an apparently organ-restricted eosinophilic disorder is instead part of systemic EGPA.

## 2 The early clinically recognizable course of EGPA

Adult-onset asthma is the most consistent clinical background of EGPA. In a multicenter respiratory cohort of 157 patients, asthma preceded EGPA diagnosis by a mean of 11.8 years, whereas peripheral eosinophilia was first documented a mean of 1.4 years before diagnosis. Asthma severity increased during the three to six months preceding systemic manifestations despite inhaled or systemic corticosteroid treatment (7). Changes in established airway disease may therefore be more informative than asthma alone. Increasing severity, corticosteroid dependence, recurrent exacerbations, persistent eosinophilia, or new extrapulmonary manifestations should prompt reconsideration of the overall phenotype.

Upper-airway disease provides another opportunity for recognition. In the same respiratory cohort, chronic rhinosinusitis occurred in 73% and nasal polyposis in 53% of patients. In a cohort specifically examining ear, nose and throat (ENT) manifestations at diagnosis, chronic rhinosinusitis with nasal polyposis was present in 52%, together with turbinate hypertrophy, nasal swelling, rhinorrhea, crusting, hearing loss, and laryngeal symptoms (8). These findings are not specific for EGPA, but a polymorphic or recurrent upper-airway phenotype associated with asthma and hypereosinophilia should strengthen clinical suspicion.

Blood eosinophilia is characteristic but strongly influenced by treatment. Repeated systemic corticosteroid courses prescribed for asthma, sinonasal disease, or other eosinophilic manifestations may transiently suppress both eosinophilia and systemic symptoms, potentially masking a multisystem phenotype and delaying recognition. A recent expert consensus proposed persistent unexplained eosinophil counts above 1,000 cells/µL in untreated patients, or above 500 cells/µL in patients previously exposed to medication likely to alter the eosinophil count, as red flags warranting assessment for EGPA in an appropriate clinical context (9). These are suspicion thresholds, not diagnostic or classification criteria.

Large cohorts have already characterized in detail the spectrum of systemic manifestations present at diagnosis. In the French Vasculitis Study Group cohort of 383 patients, asthma was present in 91.1%, peripheral neuropathy in 51.4%, ENT manifestations in 48.0%, skin lesions in 39.7%, and pulmonary infiltrates in 38.6%. Weight loss occurred in 49.3%, fever in 38.9%, myalgia in 38.9%, and arthralgia in 29.8% (6). Importantly, these cohorts largely report what was present at diagnosis but they do not establish a uniform temporal sequence.

## 3 Manifestations distributed across outpatient specialties

Before diagnosis, the EGPA phenotype may be fragmented across several outpatient settings (10). Allergy and respiratory clinics encounter adult-onset asthma, eosinophilia, and corticosteroid dependence. Otolaryngologists manage recurrent nasal polyposis, chronic rhinosinusitis, otological symptoms, or hearing impairment. Gastroenterologists investigate abdominal pain, diarrhea, dysphagia, and tissue eosinophilia. Rheumatologists and internists assess arthralgia, myalgia, weakness, inflammatory markers, or hyperCKemia (11). Dermatologists may encounter palpable purpura, urticarial or maculopapular lesions, nodules, or other vasculitic-appearing eruptions, occasionally providing an accessible site for biopsy (12). Neurologists may initially encounter paresthesia, focal weakness, pain, or isolated mononeuropathies that can resemble radiculopathy or entrapment neuropathy (13); an asymmetric, painful, multifocal pattern, particularly when evolving toward mononeuritis multiplex, should raise suspicion of vasculitic involvement (14).

Cardiac and renal involvement may occasionally be part of a more acute presentation leading to the initial recognition of EGPA (15, 16). Cardiac disease may manifest as eosinophilic myocarditis, pericardial involvement, arrhythmias, or heart failure, whereas renal involvement more commonly presents with hematuria, proteinuria, active urinary sediment, or declining renal function. These manifestations should not, however, be considered typical early outpatient red flags in the same sense as adult-onset asthma, sinonasal disease, eosinophilia, or progressively accumulating multisystem symptoms. Renal involvement is more strongly associated with MPO-ANCA positivity and a vasculitic phenotype, whereas cardiac involvement may reflect eosinophilic infiltration, vascular injury, or overlapping mechanisms (4, 17).

The practical challenge is therefore not simply to recognize individual manifestations, but to integrate their evolution over time. Repeated consultations for fatigue, weight loss, skin lesions, abdominal symptoms, muscle pain, weakness, or sensory disturbances acquire greater significance when they occur in a patient with adult-onset asthma, sinonasal disease, and eosinophilia (18). Symptoms appearing during corticosteroid reduction are especially difficult to interpret because tapering may unmask manifestations previously suppressed by treatment rather than cause new disease (19).

Clinical suspicion must remain selective. Most patients with eosinophilic asthma or nasal polyposis do not have EGPA (20). Secondary eosinophilia, hypereosinophilic syndromes, allergic bronchopulmonary aspergillosis, parasitic infections, drug reactions, and organ-restricted eosinophilic disorders remain essential differential diagnoses. Suspicion becomes more relevant when an apparently localized phenotype is accompanied by otherwise unexplained findings in additional organs (21).

## 4 Arthralgia, myalgia, and myositis

Arthralgia and myalgia are common but nonspecific manifestations. In the French cohort, myalgia and arthralgia were reported in 38.9% and 29.8% of patients, respectively (6). In the respiratory cohort, myalgia occurred in 38% and polyarthralgia in 25% (7). Despite these frequencies, their distribution, duration, inflammatory characteristics, and temporal relationship with other manifestations remain poorly defined. Moreover, creatine kinase (CK) was not systematically reported in the major cohorts, preventing estimation of how often reported myalgia reflected biochemical or structural muscle injury (22).

A distinction should therefore be made between subjective muscle pain and objective muscle involvement. Reported manifestations include diffuse myalgia, sometimes with proximal predominance, proximal weakness, focal or diffuse swelling, tenderness, and functional impairment, although a consistent anatomical distribution has not been established in EGPA. Serum CK may be normal or only mildly increased, particularly when inflammation is predominantly vascular, perivascular, or fascial rather than associated with extensive myofiber injury. Diagnostic assessment may include CK and aldolase, muscle magnetic resonance imaging (MRI) to identify muscle or fascial oedema, electromyography for myopathic changes, and biopsy in selected cases to define vascular, eosinophilic, fascial, and myofiber pathology (23). No single CK threshold distinguishes EGPA-related myositis from hereditary, metabolic, toxic, inflammatory, or other neuromuscular disorders (24). Published cases of EGPA-associated muscle involvement also show marked biochemical heterogeneity.

Evidence from vasculitic myopathy more broadly provides useful clinical context. In a recent series of 25 patients with biopsy-proven vasculitic myopathy, myopathy represented the initial manifestation of vasculitis in 80% of cases and pain was present in 76%. Notably, serum CK was elevated in only 13% of patients, whereas aldolase was elevated in 63%; electromyography showed short-duration motor unit potentials in 96% of cases, and muscle biopsy demonstrated perivascular inflammation in all specimens (25). Although this cohort was not specific to EGPA, these findings reinforce that vasculitic muscle involvement may occur despite normal or only minimally elevated CK levels.

Parent et al. described severe diffuse myalgia, weakness, fever, eosinophilia, and CK 3,708 U/L at presentation. Muscle biopsy demonstrated eosinophil-rich necrotizing vasculitis together with additional inflammatory myopathic features, including phagocytosis of non-necrotic fibers and sarcolemmal major histocompatibility complex class I (MHC-I) overexpression, although these findings are not specific and may also occur in inherited myopathies such as facioscapulohumeral and limb-girdle muscular dystrophies (26–28). Koppikar et al. subsequently reviewed biopsy-confirmed EGPA myositis and identified only seven earlier cases; their index patient had CK 2,627 U/L and developed mononeuritis multiplex after the initial myopathic presentation (22). Woo et al. reported myalgia and proximal weakness with CK 559 U/L while nerve-conduction studies were initially normal; marked axonal polyneuropathy became evident later (29). More recently, Nakagawa et al. described ANCA-negative EGPA with forearm and thigh swelling, CK 350 U/L, MRI abnormalities involving fascia and muscle, and eosinophilic infiltration of both muscle and small vessels on en bloc biopsy, despite absence of objective weakness (30).

Available reports do not establish whether EGPA-associated muscle disease represents a single pathological entity. Vasculitic myopathy, eosinophilic infiltration, fasciitis, and direct inflammatory myofiber injury have all been described, with overlapping features in some cases. Muscle involvement may be clinically appreciable at presentation, and may occasionally precede recognizable neuropathy, without necessarily preceding clinically overt vasculitic disease. Persistent myalgia associated with hyperCKemia, swelling, weakness, or imaging abnormalities therefore deserves targeted investigation.

## 5 Gastrointestinal eosinophilic disease and early EGPA recognition

Gastrointestinal involvement is reported in approximately one-fifth to one-third of patients with EGPA, although estimates vary according to cohort selection and the definition applied. Some cohorts rely mainly on symptoms attributed to EGPA, whereas others incorporate objective findings from endoscopy, imaging, histopathology, gastrointestinal bleeding, ischemia, or perforation; this heterogeneity limits direct comparison between studies. In the French cohort, gastrointestinal involvement occurred in 23.2% and abdominal pain in 20.4% (6). In the respiratory cohort, abdominal pain associated with nausea, vomiting, or diarrhea occurred in 21%, whereas gastrointestinal bleeding was less common (7).

For the purposes of this review, gastrointestinal involvement is considered objectively documented when gastrointestinal symptoms are accompanied by endoscopic, radiological, or histological abnormalities attributable to EGPA, or by major gastrointestinal complications such as bleeding, ischemia, or perforation. Isolated nonspecific gastrointestinal symptoms are therefore considered suggestive but, by themselves, insufficient to establish gastrointestinal involvement. Quantitative histopathological reporting of eosinophils per high-power field would also be desirable; however, this is not consistently provided in EGPA studies, and no validated tissue eosinophil threshold has been established specifically for EGPA-related gastrointestinal involvement. Peripheral eosinophilia may be associated with gastrointestinal involvement and has shown predictive value in some cohorts, but it cannot by itself establish tissue infiltration (31, 32).

Gastrointestinal injury may result from eosinophilic tissue infiltration, vasculitic ischemia, or both (33, 34). Endoscopic biopsies mainly sample the mucosa and may demonstrate eosinophil-rich inflammation without detecting vascular lesions located in deeper layers (35, 36). Consequently, EGPA with gastrointestinal involvement may initially be interpreted as eosinophilic gastritis, enteritis, or colitis, particularly when the respiratory and systemic history is not integrated. This diagnostic overlap is supported by case-based evidence.

Itawaki et al. reported two patients initially diagnosed with eosinophilic gastroenteritis on endoscopic biopsy; EGPA was recognized later after purpura and other systemic or vasculitic findings emerged (37). Franco et al. documented diffuse eosinophilic infiltration involving the esophagus, small bowel, and common bile duct in a patient with EGPA, illustrating that eosinophilic infiltration may extend throughout the gastrointestinal tract (38). However, available cohorts do not apply uniform diagnostic criteria for individual eosinophilic gastrointestinal disorders (EGIDs). Thus, the prevalence of standardized EGID-like phenotypes within EGPA cannot currently be estimated reliably.

The anatomical distribution of gastrointestinal involvement also deserves consideration. In a cohort of 94 patients with EGPA, 21 had gastrointestinal involvement; among these, the stomach (52.4%) and duodenum (47.6%) were the most frequently affected sites, followed by the small intestine (33.3%), colon (28.6%), and rectum (14.3%), whereas oesophageal involvement was documented in only one patient (4.8%) (32). These data refer to organ involvement rather than to standardized EGID diagnoses and therefore should not be interpreted as the prevalence of eosinophilic gastritis, enteritis, colitis, or eosinophilic esophagitis (EoE) within EGPA.

Among EGIDs, EoE is particularly relevant from an immunological perspective because it is a well-characterized type 2 inflammatory disease driven by epithelial–immune interactions and IL-4-, IL-5-, and IL-13-associated eosinophilic inflammation. This biological background also provides the rationale for targeted therapies, including dupilumab, which blocks IL-4/IL-13 signaling through IL-4Rα (39).

Evidence supporting true EoE as a distinct EGPA manifestation is particularly limited. Esophageal eosinophilic infiltration has been documented, but this does not necessarily fulfil the clinicopathological definition of primary EoE. The intended alert is therefore selective: isolated EoE does not imply EGPA, but an apparently organ-restricted EGID warrants broader clinical review when accompanied by adult-onset asthma, chronic rhinosinusitis or nasal polyposis, substantial blood eosinophilia, pulmonary infiltrates, constitutional symptoms, skin abnormalities, neuropathic complaints, arthralgia, or myalgia.

## 6 Immunopathological axes and therapeutic implications

The therapeutic landscape of EGPA partly mirrors its overlapping immunopathology. Type 2 immunity, particularly IL-5, supports eosinophil expansion, survival, and tissue activation; in parallel, B-cell autoimmunity and MPO-ANCA can promote ANCA-mediated neutrophil activation and vascular injury (40, 41). This background is clinically useful, but a strict eosinophilic-versus-vasculitic dichotomy should be avoided, because eosinophils may also infiltrate vasculitic lesions and vasculitis can occur in ANCA-negative disease.

Biological therapies directed at the IL-5 pathway primarily target the eosinophilic axis. Mepolizumab neutralizes IL-5 and demonstrated steroid-sparing efficacy in relapsing or refractory EGPA in the MIRRA trial (42). Benralizumab targets IL-5 receptor-alpha and produces profound eosinophil depletion; in the phase 3 MANDARA trial, it was noninferior to mepolizumab for remission in relapsing or refractory EGPA (43). Depemokimab is a long-acting anti-IL-5 monoclonal antibody with established clinical efficacy in severe eosinophilic asthma and sustained suppression of the eosinophilic pathway (44, 45). Its role specifically in EGPA is currently being evaluated against mepolizumab in the phase 3 OCEAN non-inferiority trial in adults with relapsing or refractory EGPA receiving standard-of-care treatment (NCT05263934) (46). Overall, therapies targeting the IL-5/IL-5R pathway predominantly address the eosinophilic component of EGPA and may contribute to control of asthma, sinonasal disease, blood eosinophilia, and glucocorticoid exposure.

For new-onset or relapsing EGPA with organ- or life-threatening manifestations, current European Alliance of Associations for Rheumatology (EULAR) recommendations support high-dose glucocorticoids combined with cyclophosphamide; rituximab may be considered as an alternative, particularly when cyclophosphamide exposure is undesirable (47). Rituximab depletes CD20-positive B cells and therefore interferes with the B-cell compartment involved in autoantibody generation and antigen presentation (48). Cyclophosphamide is a non-cell-specific alkylating agent with broad immunosuppressive effects on proliferating lymphocyte populations and should not be regarded as a selective neutrophil- or eosinophil-depleting therapy (49).

These distinctions become especially relevant before EGPA is recognized. Dupilumab blocks IL-4Rα and thereby inhibits both IL-4 and IL-13 signaling. This may reduce eosinophil trafficking from circulation into tissues without directly suppressing eosinophil production, providing a biological explanation for increases in peripheral blood eosinophil counts observed during treatment (50). Dupilumab is an established therapy for several type 2 inflammatory diseases, including asthma, chronic rhinosinusitis with nasal polyps, and EoE (51–53), but rare cases in which EGPA developed or became clinically apparent after its initiation have been described (54–56); however, these observations do not establish a direct causal relationship and should be interpreted in the context of the underlying eosinophilic disease and its natural evolution. Moreover, although it is not currently an established EGPA-directed therapy, observational experience suggests that dupilumab may be useful in selected patients with EGPA and refractory sinonasal and/or asthmatic manifestations, including patients insufficiently controlled by conventional treatment or mepolizumab (57). Concomitant treatment with dupilumab and mepolizumab has also been reported in selected refractory cases (58, 59).

The practical point is to ensure that organ-specific biologic therapy is selected within the context of the overall phenotype, in view of these potential therapeutic implications. When considering a biologic for asthma, nasal polyposis, or an EGID, clinicians should reconsider systemic EGPA when multisystem warning signs are already present. This consideration may be particularly relevant during early respiratory- or eosinophilic-dominant presentations, when the full systemic phenotype has not yet become clinically apparent.

## 7 Discussion

Available evidence indicates that several manifestations of EGPA may be clinically apparent before the disease is formally recognized, but this early interval is poorly captured by current studies. Most cohorts retrospectively record findings present at diagnosis or during the overall disease course, whereas few reconstruct their temporal sequence, treatment exposure, or cross-specialty contacts. This limitation is particularly relevant to musculoskeletal symptoms: arthralgia and myalgia occur in approximately one-quarter to two-fifths of patients, yet their objective correlates are rarely described, and major cohorts do not systematically report CK (6, 7, 22). Biopsy-proven myositis remains rare and is supported mainly by case reports.

A comparable gap affects gastrointestinal disease. Overall gastrointestinal involvement is well documented, but the prevalence of standardized EGIDs and true EoE within EGPA cannot be derived from existing cohorts. The literature more often reports symptoms, anatomical location, endoscopic lesions, or eosinophilic infiltration than uniform EGID diagnoses (37, 38). It therefore remains uncertain how often an apparently organ-restricted EGID represents the first recognized component of a broader systemic phenotype.

These findings support the clinical value of musculoskeletal and gastrointestinal manifestations as potential early clues when interpreted within a respiratory-eosinophilic and multisystem context. Their significance may increase when interpreted alongside adult-onset or increasingly difficult-to-control asthma, evolving airway disease, eosinophil counts, corticosteroid exposure, and manifestations arising in additional organs, including cardiac or renal involvement.

This multidimensional perspective is summarized in **Figure 1**, which integrates temporal evolution, organ involvement, immunopathological predominance, and clinical setting within a single conceptual model. This approach is particularly relevant in ambulatory practice, where early manifestations may emerge across different specialties and remain clinically disconnected until their temporal and multisystem relationships are recognized. Patients with still-unrecognized EGPA may be encountered not only in vasculitis referral centers but in everyday allergy, respiratory, ENT, gastroenterology, rheumatology, dermatology, neurology, and primary-care clinics, where individual components of the disease may initially appear unrelated. Recognition depends on reconstructing the clinical timeline, reviewing historical eosinophil counts and corticosteroid exposure, and reconsidering organ-specific diagnoses when additional manifestations accumulate over time.

**Figure 1:**
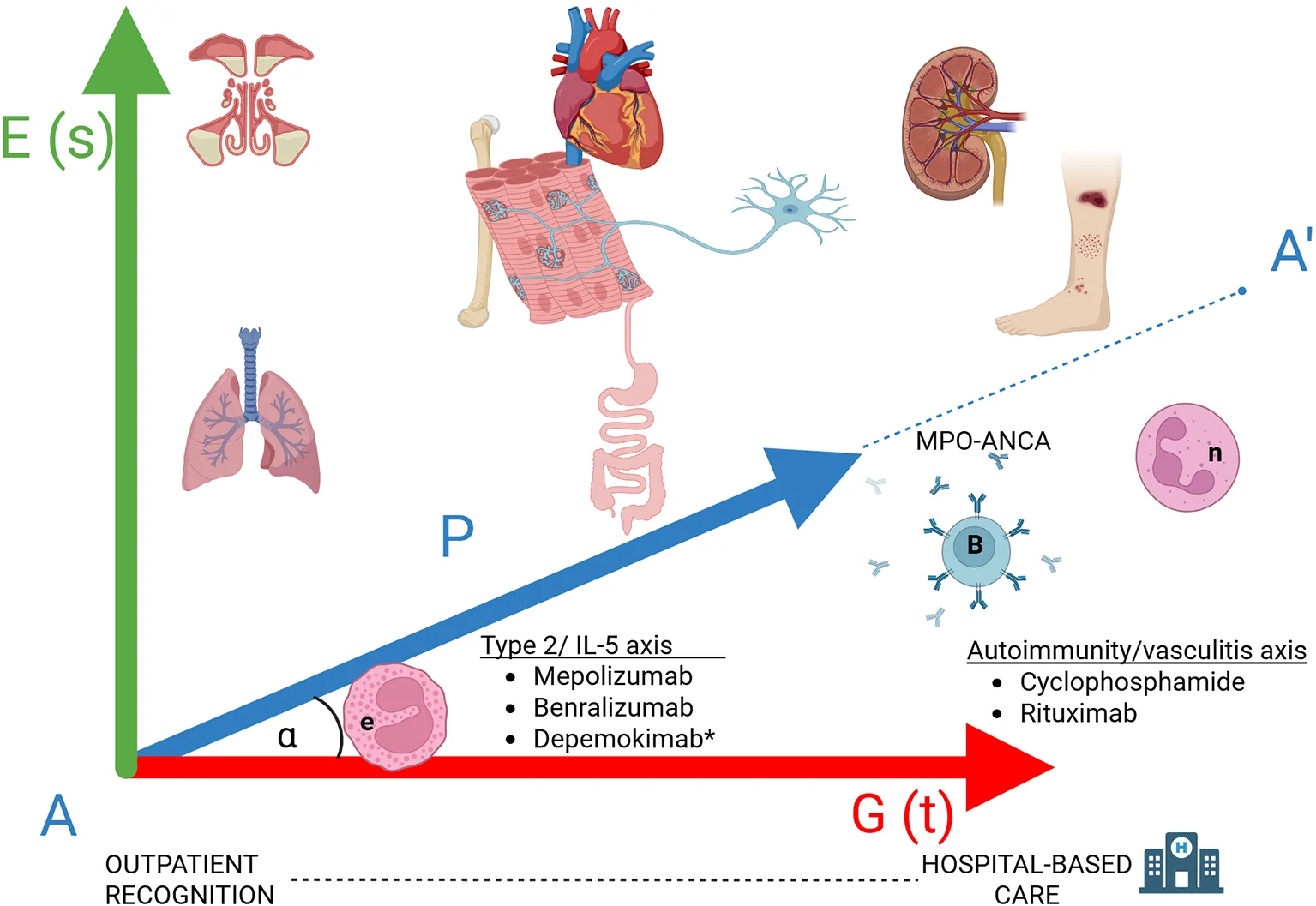
A multidimensional conceptual model of EGPA. The ordinate axis E, expressed as a function of the spatial extent of organ involvement (s), and the abscissa axis G, expressed as a function of disease evolution over time (t), define a Cartesian plane in which the patient and the extent of visceral involvement can be positioned hic et nunc, at any given point in the disease course. From this plane arises a conceptual progression velocity vector (P), extending along a longitudinal dimension beyond the Cartesian plane. The origin of the axes is represented by point A (Allergy), where allergic and respiratory prodromal manifestations predominate, whereas the opposite conceptual pole A′ (Autoimmunity) represents increasing predominance of autoimmune and vasculitic mechanisms. A and A′ represent conceptual poles of immunopathological predominance rather than mandatory sequential stages, as eosinophilic and vasculitic mechanisms may overlap throughout the disease course. The partly dashed ray connecting A and A′ defines the immunopathologic trajectory of eosinophilic granulomatosis with polyangiitis (EGPA). The angle α formed between the G axis and the P vector encompasses the eosinophilic domain, with the eosinophil (e) positioned as a bridging element between early allergic manifestations and clinically overt eosinophilic tissue-infiltrative disease. This domain is characterized by type 2 inflammation and IL-5-dependent eosinophilic biology and is therefore therapeutically targeted by anti-IL-5/IL-5R agents such as mepolizumab, benralizumab, and depemokimab*. By contrast, the right-hand portion of the triangle beneath the blue vector represents the more vasculitic/autoimmune-dominant domain of EGPA, characterized by B lymphocytes (B), myeloperoxidase-specific anti-neutrophil cytoplasmic antibodies (MPO-ANCA), neutrophils (n), and ANCA-mediated neutrophil activation. Therapeutic strategies more typically used in this context include cyclophosphamide and rituximab. The upper triangle provides a schematic representation of organ involvement across the disease course. Rhinosinusal and pulmonary manifestations are positioned toward the left, reflecting their frequent association with the allergic/eosinophilic phenotype. Cardiac, gastrointestinal, and musculoskeletal involvement occupy an intermediate position, as they may reflect overlapping mechanisms of inflammation, eosinophilic tissue infiltration, and vasculitis. These manifestations may coexist with, or in some cases precede, neurological involvement, particularly mononeuritis multiplex, which more strongly reflects a vasculitic phenotype. The neural representation therefore leads toward the right-hand side of the image, where other characteristic vasculitic manifestations, including palpable purpura and renal involvement, are depicted. Finally, the image also illustrates a shift in the predominant clinical setting across this multidimensional disease trajectory, from outpatient recognition of early allergic, respiratory, and eosinophilic manifestations toward an increasing likelihood of hospital-based assessment and care in patients with more overt systemic or vasculitic involvement. The spatial position of individual manifestations is schematic and reflects predominant clinical and immunopathological associations rather than mutually exclusive disease phases. *Depemokimab is a long-acting anti-IL-5 monoclonal antibody currently under investigation in EGPA in the phase 3 OCEAN trial (NCT05263934). Created in BioRender. Ginaldi, (L) (2026). https://BioRender.com/xfzsvj3.

The therapeutic consequences further reinforce the need for accurate phenotyping, because therapies directed at eosinophilic inflammation, B-cell autoimmunity, broad immunosuppression, or other type 2 pathways have distinct therapeutic roles according to disease phenotype and severity.

## 8 Conclusions

EGPA may become clinically recognizable in outpatient settings before its full multisystem phenotype is evident. Adult-onset asthma, particularly when progressively worsening or difficult to control, together with sinonasal disease and eosinophilia, may acquire greater diagnostic relevance when accompanied over time by otherwise unexplained extrapulmonary manifestations, including musculoskeletal, gastrointestinal, cutaneous, neurological, cardiac, or renal involvement.

The principal practical message concerns both clinical setting and temporal evolution. Findings documented across separate outpatient specialties may become more informative when interpreted longitudinally and within a systemic context. Recognizing an evolving multisystem pattern can support timely diagnostic assessment and more appropriate treatment selection according to the overall EGPA phenotype.
